# Progress and priorities in reducing the time to cancer diagnosis

**DOI:** 10.1038/s41416-022-02045-5

**Published:** 2022-11-07

**Authors:** B. D. Nicholson, G. Lyratzopoulos

**Affiliations:** 1 Academic Clinical Lecturer and Cancer Research Theme Lead, Nuffield Department of Primary Care Health Sciences, University of Oxford, OX26GG Oxford, UK; 2 Professor of Cancer Epidemiology and Lead of Epidemiology of Cancer Healthcare and Outcomes (ECHO) Group, University College London, 1-19 Torrington Place, WC1E 7HB London, UK

**Keywords:** Diagnosis, Signs and symptoms, Cancer epidemiology

## Abstract

Key developments in early diagnosis research and policy since the publication of the highly cited BJC review “Is increased time to diagnosis and treatment associated with poorer outcomes?” by Neal et al. in 2015 are summarised. Progress achieved since 2015 is described and priorities for further research identified.

The systematic review by Neal et al. “Is increased time to diagnosis and treatment associated with poorer outcomes” published in the 2015 early diagnosis BJC supplement has become one of the most highly cited papers in the field with 470 Web of Science citations by September 2022 [1]. While Neal et al. dissect methodological shortcomings in the evidence they have reviewed, they posit that shortening the time to cancer diagnosis and treatment does convey prognostic benefits, particularly for certain cancers and patient groups [1]. Building on this conclusion, we overview key developments in policy and evidence for early diagnosis that have ensued since the publication of this seminal paper.

The publication of the NICE guidelines ‘Suspected cancer: Recognition and referral” in the same year as the Neal et al. review (2015) represents an important milestone in efforts to improve diagnosis of as-yet-undiagnosed cancer in symptomatic patients [2]. A unique feature of these guidelines is the endorsement of an explicit 3% cancer risk threshold at which patients ought to be investigated. Like all social value judgements, this threshold represents the assessment of benefits, risks, and cost-effectiveness of diagnostic technologies available at that time. We now know that it may be possible to lower referral thresholds for some patients without being overly demanding of resources, whilst effectively increasing them when positive triage tests identify higher-risk symptomatic patients [3]. There has been a continuous and inexorable rise in urgent referrals for suspected cancer since 2009-10. Using the latest reported data (12-month period to July 2022), there are more than 2.7 m referrals for suspected cancer each year in England. The increasing proportion of cancer patients detected via this route has been credited for welcome reductions in diagnoses through emergency presentation, associated with worse prognosis [4]. However, these positive developments come at a cost as only around one in 15 patients referred is found to have cancer [5]. For cancers with few, variable, or low-risk symptoms and no primary care tests, there are still limited options to improve early diagnosis.

Nearly half of symptomatic patients who will go on to be diagnosed with cancer do not present with red-flag symptoms included in guidelines [6]. Guidelines may be surpassed if clinicians assess patients to be at-risk of undiagnosed cancer despite not meeting formal criteria for specialist investigation or assessment. Research into GP “gut-feeling” emphasised the importance of subtle cues and clinical observations not easily incorporated into guideline recommendations or decision-support tools [7]. Offering an expedited referral route for these patients is appropriate but it remains a challenge for clinicians to communicate what led to their higher index of suspicion when making referrals. Conversely, non-adherence to referral recommendations for “red-flag” symptoms also occurs. A recent study reported that the risk of cancer among patients with red-flag symptoms not promptly referred was a third of that observed for referred patients with the same symptoms [8]. Whilst this study indicates that guideline non-adherence may be driven by risk-based patient selection, this assessment seems imperfect as the residual cancer risk remains high [8]. Therefore, there is a need for both widening referral criteria and improving adherence to them, supported by new evidence to guide patient selection.

Given the high proportion of cancer patients without typical symptoms, attention is being paid to presentations not obviously associated with a body organ or system (e.g. loss of weight, fatigue, abdominal pain), and to creating care pathways and services that can help to achieve rapid diagnostic resolution in these patients. Research on the positive predictive value of symptoms has determined appropriate risk periods following presentation to avoid inflating risk estimates when using inappropriately long follow-up periods that incorporate background incidence unrelated to presenting symptoms [9]. Dedicated non-specific symptoms pathways have been introduced in the UK emulating similar services in Denmark to support investigations for suspected cancer across different body organs or systems. Early evaluations demonstrate diagnostic yields for cancer that are higher than pathways for specific symptoms, supporting the sustainability of this route to the diagnosis of less common cancers or serious non-malignant disease in a third of referred patients [10]. As this broad investigative approach can also detect findings of uncertain clinical significance that require further monitoring, health economic analyses, longer-term follow-up of patient reported outcomes, and further diagnostic test development are needed to help guide future development of these policies.

A rapid development ensuing the publication of the Neal et al. 2015 review, further accelerated by the need for non-invasive community-based risk stratification during the COVID-19 pandemic, is the implementation of Faecal Immunochemical Testing (FIT) as a triage test for patients visiting primary care with lower gastrointestinal symptoms. The success of this simple test, which is now backed by ample evidence for its effectiveness in symptomatic patients in primary care [11], highlights the considerable gap in similar community-based diagnostics to support patient triage in primary care. Substantial progress has been made to understand the risks associated with abnormalities in commonly used blood tests (e.g. raised inflammatory markers, thrombocytosis) and the combination of blood test abnormalities with presenting symptoms (e.g. raised ESR and low haemoglobin in suspected myeloma, simple blood test combinations in weight loss) [12, 13]. Age-adjusted thresholds for CA125 values in symptomatic women offer a more precise approach to patient selection for investigation for possible ovarian cancer [14]. Research is underway to understand whether incorporating biomarker change over time adds to the predictive value of the test result closest to a symptomatic presentation [15]. Gaps in the availability of primary care-based diagnostic technologies can also be ameliorated by the smarter deployment of existing specialist tests (such as direct access to CT Chest for suspected lung, and to mpMRI as adjunct to PSA testing for prostate cancer), though substantial technological innovation and additional infrastructure including community-based hubs to host community-based imaging services are needed.

Lowering haemoglobin detection thresholds in colorectal cancer screening using FIT, innovations in risk-stratified CT-based lung cancer screening, and cervical self-sampling represent obvious improvements in asymptomatic detection. Improvements in the effectiveness and delivery of the bowel cancer screening programme also contribute to reducing emergency presentations from colorectal cancer [16]. Yet for many cancers, often with poor prognosis (e.g. pancreas) there has long been no suitable candidate screening test. Non-invasive multi-cancer early detection (MCED) blood testing based on cancer-specific genetic alterations in circulating tumour DNA offers a possible route to the earlier identification of these cancers. Varied MCED performance by cancer type and stage, and the increasing range of MCED technologies being brought to market, mean that investment in large-scale prospective evaluations is vital to match the MCED to the most appropriate population and cancers [17]. MCED tests could hold promise in aiding risk stratification in symptomatic patients selecting some for urgent investigation or identifying those in whom urgent referral can be substituted by alternative management. Clinician and public preferences will be influenced by the pace of innovation, associated costs, test accuracy, and the cascades of further investigations needed following positive tests. Robust evaluations of these promising technologies will require resources and time to guide optimal implementation.

Considering health system factors, international comparisons suggest that far from being a ‘UK issue’ the problem of prompt cancer diagnosis is ubiquitous across contemporary health systems, including the US and other high-income countries, though the same underlying problem is manifested differently depending on health service organisation, healthcare professional cultures, and the public understanding of cancer [18–20]. Inequalities persist in key measures and markers of early diagnosis, such as emergency presentation and stage at diagnosis [21, 22]. Increasingly we appreciate that multiple factors influence the quality and speed of the diagnostic process beyond the diagnostic skills of individual clinicians, such as the tests and services available to them, time constraints to consultation duration and the quality of doctor-patient communication [23, 24]. An approach to improving diagnosis is to bolster the resilience of the diagnostic process by proactive patient follow-up, robust tracking of the results of ordered tests, involvement of more than one clinician in difficult cases, and patient empowerment through safety-netting [25, 26]. Such a’systems’ approach may not prevent delays from occurring but could identify them early to minimise their length and possible associated psychological or prognostic harm.

Do these significant advances that followed the Neal et al. review match their call for “well-designed and well-analysed prospective studies” to “understand the likely effect of interventions” and to “inform the development of targeted intervention studies, to improve outcomes”? Innovations in patient selection, testing, and pathway configuration are often deployed with little supportive evidence. To address the call made by Neal et al. for better evidence to support and target interventions we must couple innovation and real-world implementation with evaluation, ideally encompassing experimental designs to ensure clinical impact and equitable translation into practice. To generate the robust evidence to guide further improvements, sustained investment in health system analytics will be needed to establish whether interventions result in shorter intervals from symptom to diagnosis, better treatment and outcomes for cancer patients.
